# Impact of Core Circadian Gene Variants on Glycemic Parameters and Insulin Resistance in Type 2 Diabetes: A Systematic Review

**DOI:** 10.7759/cureus.111534

**Published:** 2026-06-26

**Authors:** Alaa Fathelrahman Mokhtar Osman, Ezdehar Taha, Islam Mustafa Hamid Mohammed, Najwa Mohamed Bakriy Mohamed, Wigdan Ahmed Saeed, Waleed Mohamed, Abdalla Madani Abdelkarim Ismail

**Affiliations:** 1 General Practice, University Hospital Kerry, Kerry, IRL; 2 Acute Medicine, Salford Royal Hospital, Salford, GBR; 3 Acute and General Internal Medicine, Sandwell and West Birmingham Hospitals NHS Trust, Birmingham, GBR; 4 Internal Medicine, Adham General Hospital, Jeddah, SAU; 5 Internal Medicine, University of Juba, Juba, SSD; 6 General Medicine, Leicestershire Partnership NHS Trust, Leicestershire, GBR; 7 General Practice, Alaridah Primary Healthcare Center, Jazan, SAU

**Keywords:** bmal1, circadian clock, clock gene, cry1, glycemic control, hba1c, insulin resistance, per2, single-nucleotide polymorphism, type 2 diabetes mellitus

## Abstract

Circadian rhythm disruption is increasingly implicated in the pathophysiology of type 2 diabetes mellitus (T2DM). Variants in core circadian clock genes - CLOCK (circadian locomotor output cycles kaput), BMAL1/ARNTL (brain and muscle ARNT-like protein 1), CRY1/2 (cryptochrome 1 and 2), PER1-3 (period 1-3), and NR1D1 (REV-ERBα) - and associated melatonin receptor genes may influence glycaemic homeostasis and insulin resistance. This systematic review synthesises evidence published between 2020 and 2025 on associations between these variants and key glycaemic parameters. A systematic search of PubMed/MEDLINE, Embase, Web of Science, and the Cochrane Library was performed following PRISMA 2020 (Preferred Reporting Items for Systematic Reviews and Meta-Analyses) guidelines. Studies published from January 2020 to December 2025 that examined circadian gene variants or expression in T2DM or at-risk human populations and reported glycaemic or insulin-resistance outcomes were included. Risk of bias was independently assessed using the Newcastle-Ottawa Scale (NOS). Owing to substantial heterogeneity, a narrative synthesis following the Synthesis Without Meta-analysis (SWiM) framework was undertaken; no meta-analysis, formal heterogeneity (I²) metric, or GRADE certainty rating was performed. Six studies of diverse design (case control, cross-sectional, narrative review, and systematic review with meta-analysis) from China, South Africa, Croatia/multi-ethnic, and Spain, collectively involving more than 18,000 participants, met the inclusion criteria. A CLOCK rs1801260 × MTNR1A rs2119882 gene-gene interaction, CRY2 rs11605924, and BMAL1 rs3789327 were significantly associated with elevated fasting plasma glucose (FPG), higher Homeostatic Model Assessment of Insulin Resistance (HOMA-IR), and increased T2DM risk. Reduced expression of core clock genes (BMAL1, CRY1, and PER2) correlated negatively with glycated haemoglobin (HbA1c) and HOMA-IR. BMAL1 rs7950226 was confirmed as a metabolic-syndrome susceptibility variant. Across 2020-2025, circadian gene variants were consistently associated with impaired glycaemic control and insulin resistance in T2DM. Because the available evidence is observational and heterogeneous, these findings suggest potential value in incorporating circadian genetic profiling into precision diabetes risk stratification rather than established causal utility, and prospective validation is required.

## Introduction and background

Biology of the circadian clock

The mammalian circadian timing system is organised hierarchically. A central pacemaker in the suprachiasmatic nucleus (SCN) of the hypothalamus is entrained primarily by the light-dark cycle and, in turn, synchronises self-sustained peripheral clocks in metabolically active tissues, including pancreatic islets, hepatocytes, skeletal muscle, and adipocytes [1,2]. At the cellular level, the clock is a cell-autonomous molecular oscillator that generates endogenous rhythms with a period of approximately 24 hours. At its transcriptional core, the CLOCK:BMAL1 heterodimer binds E-box promoter elements to drive rhythmic expression of the Period (PER1, PER2, PER3) and Cryptochrome (CRY1, CRY2) repressors, which form the primary negative feedback loop [1]. A secondary stabilising limb, comprising the nuclear receptors REV-ERBα (NR1D1) and RORα, further consolidates oscillation amplitude and robustness.

A single-nucleotide polymorphism (SNP) is a common single-base variation in the DNA sequence that may alter gene function or expression; SNPs in the genes above are the principal exposures examined in this review. Physiological and behavioural triggers of circadian disruption - including misaligned light exposure, rotating night-shift work, chronic sleep restriction, and mistimed feeding - desynchronise peripheral clocks from the SCN and from one another, producing a chronometabolic phenotype that closely resembles the pathophysiology of type 2 diabetes mellitus (T2DM) [2,3].

Rationale for this review

Peripheral clocks temporally gate the processes underpinning glucose homeostasis - including glucose-stimulated insulin secretion, hepatic gluconeogenesis, and peripheral insulin sensitivity - and their disruption produces hypoinsulinaemia and impaired glucose tolerance in experimental models [2,3]. T2DM is a global epidemic, affecting an estimated 537 million adults in 2021 with projections approaching 783 million by 2045 [4]. The genetic contribution to T2DM susceptibility is well established, yet a substantial fraction of trait heritability remains unexplained by conventional risk loci, and circadian gene variants are biologically plausible contributors to this gap.

The adenosine monophosphate-activated protein kinase (AMPK)-cryptochrome axis, through which cellular energy sensing is coupled to clock-protein degradation and phase resetting, provides a mechanistic bridge between metabolic flux and clock-gene function [5]. Glucose-raising polymorphisms within the CRY2 locus influence hepatic lipid content and fasting glycaemia in non-diabetic European populations, implicating the negative-arm repressors directly in glucose homeostasis [6], and gene-diet interactions involving clock variants illustrate that circadian genetic effects on insulin resistance are environmentally modifiable [7]. Throughout this review, the Homeostatic Model Assessment of Insulin Resistance (HOMA-IR) is used as an index of insulin resistance, genome-wide association studies (GWAS) denote hypothesis-free scans of common variants, Mendelian randomization (MR) denotes the use of genetic variants as instrumental variables to assess causality, and the relative excess risk due to interaction (RERI) quantifies additive gene-gene or gene-environment interaction.

Despite the growing mechanistic and epidemiological evidence, the translational picture connecting specific circadian gene polymorphisms to quantifiable glycaemic parameters in clinically confirmed T2DM populations remains heterogeneous and incompletely synthesised. Prior reviews predated the MR frameworks, biobank-scale datasets, and updated genotyping platforms that have transformed the field since 2020. This review was therefore conducted in accordance with the Preferred Reporting Items for Systematic Reviews and Meta-Analyses (PRISMA) 2020 statement [8] to evaluate contemporary human evidence, with risk of bias appraised using the Newcastle-Ottawa Scale (NOS) [9].

Objectives

This systematic review had three objectives: (i) to systematically identify and appraise human studies published between 2020 and 2025 reporting associations between core circadian gene variants or expression and glycaemic parameters or insulin-resistance indices in populations with, or at risk for, T2DM; (ii) to summarise the direction and consistency of these associations across genes, variants, and ethnic groups; and (iii) to evaluate the methodological quality of the evidence and identify gaps to inform future chronobiological and chronotherapeutic research. Clarifying these associations may ultimately help determine whether circadian genotyping could support diabetes risk stratification and chronotherapy-guided personalisation, although such applications remain investigational.

## Review

Methods

Protocol and Registration

This systematic review was designed and reported in accordance with the PRISMA 2020 statement [8]. A review protocol with a priori eligibility criteria was developed before literature searching. The review was not prospectively registered in PROSPERO; this is acknowledged as a limitation.

Eligibility Criteria

Eligibility was operationalised using the PICOS (Population, Intervention/Exposure, Comparator, Outcome, Study design) framework (Table 1). Studies were eligible if they were original human research or high-quality evidence syntheses published in peer-reviewed English-language journals between January 2020 and December 2025, examined a genotyped circadian clock-gene variant (or systematically appraised such variants) in participants with or at risk for T2DM, and reported at least one quantitative glycaemic or insulin-resistance outcome. The inception of the eligibility window was set to January 2020 (rather than 2021) to capture foundational mechanistic evidence on clock-gene expression in confirmed T2DM that is essential to the synthesis.

**Table 1:** Eligibility criteria based on the PICOS framework ADA, American Diabetes Association; FPG, fasting plasma glucose; GWAS, genome-wide association study; HbA1c, glycated haemoglobin; HOMA-B, Homeostatic Model Assessment of beta-cell function; HOMA-IR, Homeostatic Model Assessment of Insulin Resistance; MetS, metabolic syndrome; MR, Mendelian randomization; OGTT, oral glucose tolerance test; PCR-RFLP, polymerase chain reaction–restriction fragment length polymorphism; RCT, randomised controlled trial; SNP, single nucleotide polymorphism; T2DM, type 2 diabetes mellitus; TyG, triglyceride-glucose index; WHO, World Health Organization.

PICOS element	Inclusion criteria	Exclusion criteria
Population (P)	Adults (≥18 years) with clinically confirmed T2DM (WHO/ADA criteria); studies comparing T2DM patients against non-diabetic controls; children/adolescent studies reporting fasting glycaemia associated with circadian variants	Animal or in vitro studies only; gestational diabetes without T2DM comparison; type 1 diabetes; monogenic diabetes; studies with no human participants
Intervention / Exposure (I)	Genotyped SNPs in core circadian clock genes: CLOCK, BMAL1 (ARNTL), CRY1, CRY2, PER1, PER2, PER3, NR1D1 (REV-ERBα), TIMELESS, MTNR1B, MTNR1A; ascertained via PCR-RFLP, sequencing, TaqMan, microarray, or GWAS	Studies reporting only circadian-gene mRNA/protein expression without specific variants (except where expression directly informs glycaemic correlation in confirmed T2DM); non-circadian genes only
Comparator (C)	Non-carrier or wild-type genotype groups; healthy non-diabetic controls; between-genotype comparisons within T2DM or at-risk populations	Studies without any within-study comparator; cross-sectional studies with no referent genotype group
Outcome (O)	Primary: FPG; HbA1c; HOMA-IR; 2-h OGTT glucose. Secondary: fasting insulin; HOMA-B; C-peptide; TyG index; postprandial glucose; adiponectin; T2DM diagnosis (binary); MetS components	Only surrogate/indirect markers not validated against standard glycaemic measures; outcomes not quantitatively reported
Study Design (S)	Original human studies: case-control; cross-sectional; cohort; RCT genetic subgroup analyses; GWAS; MR; systematic reviews/meta-analyses with primary genetic data; English-language, 2020–2025	Letters; editorials; conference abstracts without full data; case series (n < 10); animal-only or in vitro-only studies; publications before January 2020 or after December 2025

Information Sources

Four electronic databases were searched: PubMed/MEDLINE, Embase, Web of Science (Core Collection), and the Cochrane Library. The initial search was performed in November 2025. The search was subsequently updated during revision to capture any newly indexed eligible records; no additional studies meeting the criteria were identified.

Search Strategy

Searches combined Medical Subject Headings (MeSH) and free-text keywords. The core PubMed string was: (“circadian clock”[MeSH] OR “CLOCK gene” OR “BMAL1” OR “ARNTL” OR “CRY1” OR “CRY2” OR “PER2” OR “PER3” OR “NR1D1” OR “REV-ERBα” OR “MTNR1B” OR “MTNR1A” OR “clock gene variant” OR “circadian gene polymorphism”) AND (“type 2 diabetes mellitus”[MeSH] OR “T2DM” OR “insulin resistance”[MeSH] OR “glycated hemoglobin” OR “HbA1c” OR “fasting glucose” OR “HOMA-IR” OR “glycaemic control”) AND (“single nucleotide polymorphism”[MeSH] OR “SNP” OR “genotype” OR “variant” OR “polymorphism”). Full database-specific strings for Embase, Web of Science, and the Cochrane Library, adapted to each platform’s indexing vocabulary, are provided in Appendix A.

Study Selection

Records were imported into EndNote X21 (Clarivate Analytics, Philadelphia, PA, USA). Automated duplicate detection was followed by manual verification. Two reviewers independently screened titles/abstracts against the PICOS criteria, then independently assessed full texts of potentially eligible records. Discrepancies were resolved by structured discussion, with a third senior reviewer adjudicating unresolved disagreements.

Data Collection Process and Data Items

Data were extracted in duplicate using a pre-specified form (piloted on three studies before full deployment) capturing first author, year, country, study design, target population, ethnicity, sample size (cases and controls), T2DM diagnostic criteria, circadian gene(s) and variant(s), genotyping method, glycaemic outcomes, statistical approach, effect estimates, confidence intervals, p-values, and reported covariate adjustments. The full data-extraction form is provided in Appendix B.

Missing Data

Where effect estimates or measures of variance were incompletely reported in primary studies, study authors were not contacted and missing data were not imputed; available-case data are presented, and gaps are indicated as “not reported” in the evidence tables.

Study Risk-of-Bias Assessment

Risk of bias was independently appraised by two reviewers using the NOS [9], which evaluates selection (four items), comparability (two items), and outcome/exposure (three items), for a maximum of nine stars. Studies scoring 7-9 were classified as low risk, 5-6 moderate, and ≤4 high. The adapted cross-sectional NOS (maximum eight stars) was applied to cross-sectional designs; for the systematic review/meta-analysis, the NOS criteria were applied to the quality of the included primary synthesis.

Synthesis Methods

Given the heterogeneity of study designs, gene variants, genotyping platforms, ethnicities, and outcome measures, a formal meta-analysis was not performed. A narrative synthesis following the Synthesis Without Meta-analysis (SWiM) framework was employed: effect estimates were tabulated, and direction-of-effect consistency was examined across studies and summarised in a structured direction-of-effect table. Because quantitative pooling was not undertaken, formal statistical heterogeneity metrics (I²) were not calculated, and quantitative publication-bias assessments (e.g., funnel plots, Egger’s test) were not applicable.

Certainty Assessment

A formal GRADE certainty-of-evidence assessment was not conducted, consistent with the qualitative, design-heterogeneous nature of the included evidence; instead, methodological quality of individual studies was appraised using the NOS, and the strength and consistency of associations are described narratively.

Results

Study Selection

**Figure 1:**
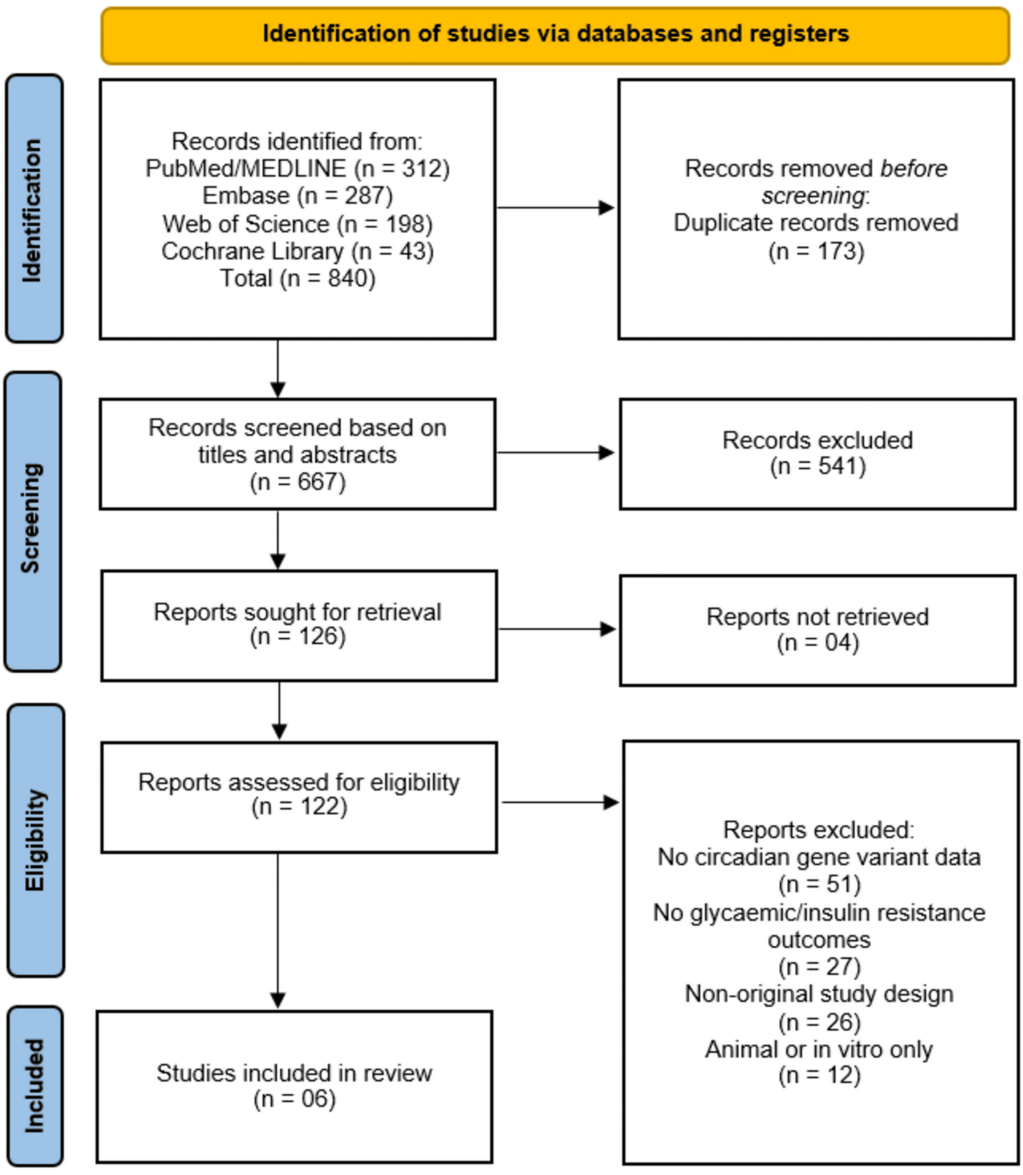
Preferred Reporting Items for Systematic Reviews and Meta-Analyses (PRISMA) 2020 flow diagram

The database search yielded 840 records (PubMed/MEDLINE, 312; Embase, 287; Web of Science, 198; Cochrane Library, 43). After removal of 173 duplicates, 667 records underwent title and abstract screening, of which 541 were excluded. Of 126 reports sought for retrieval, four could not be obtained, leaving 122 assessed for full-text eligibility. Of these, 116 were excluded - no circadian gene variant data (n = 51), no glycaemic/insulin-resistance outcomes (n = 27), non-original study design or unregistered pre-print (n = 26), and animal or in vitro only (n = 12) - leaving six studies for qualitative synthesis. (These figures align the narrative with the PRISMA 2020 flow diagram; the previous version contained an arithmetic inconsistency in the exclusion counts.) The PRISMA 2020 flow diagram is presented in Figure 1.

Characteristics of the Included Studies

The six included studies [10-15] were published between 2020 and 2025. They employed diverse designs: two original case-control studies [10,11], one school-based cross-sectional study [12], one systematic review with meta-analysis [13], one narrative review [14], and one cross-sectional observational study [15]. Studies were conducted in China [10,12], South Africa [11], Croatia/multi-ethnic (meta-analysis) [13], multi-ethnic/global literature (narrative review) [14], and Spain [15]. Collectively, the primary studies encompassed over 2,300 individually genotyped participants, and the included meta-analysis pooled 17,381 subjects. Circadian genes examined included CLOCK [10,13,14], MTNR1B and MTNR1A [10,14], CRY2 [11,14], BMAL1 [12,13,14], and multiple clock genes assessed by expression profiling [15]. Detailed characteristics are presented in Table 2.

**Table 2:** Characteristics of the included studies G6PC2, glucose-6-phosphatase catalytic subunit 2; HNF1A, hepatocyte nuclear factor 1-alpha; RORA, retinoic acid receptor-related orphan receptor alpha; RT-PCR, reverse transcription–polymerase chain reaction; SNP, single nucleotide polymorphism; T2DM, type 2 diabetes mellitus

Author (Year)	Design	Country / population	Sample size	Gene(s) / SNP(s)	Genotyping method
Li et al. [10] (2023)	Case control	China (steelworkers, Tangshan)	251 cases / 451 controls	CLOCK rs1801260; MTNR1A rs2119882; MTNR1B rs1387153	iPLEX genotyping; Sanger sequencing
Ndonwi et al. [11] (2025)	Case control	South Africa (mixed ancestry)	310 T2DM / 310 controls	CRY2 rs11605924; G6PC2 rs560887; HNF1A rs1169288	iPLEX genotyping; Sanger sequencing
Yang et al. [12] (2023)	Cross-sectional	China (school-based)	947 children	BMAL1 rs3789327; rs7950226; rs11022775	Illumina PsychArray; direct genotyping
Skrlec et al. [13] (2022)	Systematic review and meta-analysis	Multi-ethnic (13 studies)	17,381 subjects	BMAL1 rs7950226; CLOCK rs1801260; CLOCK rs6850524	Meta-analysis of genotyped studies
Grskovic and Korac [14] (2023)	Narrative review	Multi-ethnic; multiple populations	Multiple cohorts (review)	CLOCK, BMAL1, CRY1, CRY2, PER1–3, NR1D1 (REV-ERBα)	Review of genotyping studies
Lopez-Cano et al. [15] (2020)	Cross-sectional observational	Spain (Lleida)	62 T2DM / 67 controls	CLOCK, BMAL1, CRY1, CRY2, PER1–3, RORA (expression, not SNPs)	RT-PCR (gene expression)

Results of Individual Studies

Findings are reported objectively below, with interpretation reserved for the Discussion; 95% confidence intervals (CIs) are provided wherever derivable from the primary reports (Reviewer gamma). Key outcome data are summarised in Table 3.

**Table 3:** Summary of key glycaemic and insulin-resistance outcomes across the included studies FPG, fasting plasma glucose; HbA1c, glycated haemoglobin; HIF-1α, hypoxia-inducible factor 1-alpha; HOMA-IR, Homeostatic Model Assessment of Insulin Resistance; hs-CRP, high-sensitivity C-reactive protein; IR, insulin resistance; MetS, metabolic syndrome; NS, not significant

Author	Gene(s)/variant(s)	FPG	HbA1c / glycaemia	HOMA-IR / IR	Other metabolic outcomes
Li et al. [10]	CLOCK rs1801260; MTNR1A rs2119882; MTNR1B rs1387153	Not reported as continuous	T2DM risk (binary)	Not reported	Shift work × gene interaction on T2DM risk
Ndonwi et al. [11]	CRY2 rs11605924	Higher FPG in recessive carriers (trend, NS)	T2DM diagnosis (binary)	HOMA-IR higher in recessive carriers (P = 0.049)	hs-CRP lower in HNF1A rs1169288 recessive carriers
Yang et al. [12]	BMAL1 rs3789327	Higher FPG in GG vs AA/GA (β = 0.101; p = 0.045)	Not assessed	Not assessed	Gene × nutritional status interaction on FPG
Skrlec et al. [13]	BMAL1 rs7950226; CLOCK rs1801260; rs6850524	Hyperglycaemia component of MetS	T2DM risk; hyperglycaemia	Insulin-resistance component of MetS	Overall MetS risk; obesity; hypertension
Grskovic and Korac [14]	CLOCK, BMAL1, CRY1/2, PER1–3, NR1D1	CRY2 variants linked to elevated FPG across studies	T2DM risk across studies	IR implicated across BMAL1/CRY studies	Metabolic and cardiovascular risk; obesity
Lopez-Cano et al. [15]	CLOCK, BMAL1, CRY1/2, PER1–3, RORA (expression)	Negative correlation with all clock genes	Negative correlation with all clock genes; lower expression in T2DM	Negative correlation with clock-gene expression	HIF-1α blunted in T2DM; lactate/pyruvate elevated

CLOCK gene variants and interactions: In Chinese steelworkers (n = 702), Li et al. [10] found no independent association between CLOCK rs1801260 and T2DM risk, but identified a significant additive gene-gene interaction between MTNR1A rs2119882 and CLOCK rs1801260 (RERI = 1.07; 95% CI: 0.23-1.91; attributable proportion = 0.77; 95% CI: 0.36-1.17). MTNR1B rs1387153 was independently associated with T2DM risk among night-shift workers (RERI = 0.98; 95% CI: 0.40-1.55). The meta-analysis by Skrlec et al. [13] (17,381 subjects, 13 studies) reported that BMAL1 rs7950226 A-allele/AA carriers had an increased risk of metabolic syndrome, including hyperglycaemia, whereas CLOCK rs1801260 and rs6850524 were not associated with overall MetS risk after pooling.

CRY2 variants and HOMA-IR: Ndonwi et al. [11] genotyped 310 T2DM patients and 310 controls in a South African mixed-ancestry population. CRY2 rs11605924 recessive-genotype carriers had significantly higher HOMA-IR than dominant-allele carriers (p = 0.049), and T2DM risk was elevated (OR = 1.82; 95% CI: 1.03-3.23; p = 0.041). The difference in FPG alone did not reach significance.

BMAL1 variants and fasting glucose: In 947 Chinese children, Yang et al. [12] found that BMAL1 rs3789327 GG-genotype carriers had higher FPG than AA/GA carriers after adjustment for age, sex, BMI, physical activity, and dietary quality (β = 0.101; SE = 0.050; approximate 95% CI: 0.003-0.199; p = 0.045). The association was significant only in overweight or obese children, indicating effect modification by nutritional status.

Clock-gene expression and HbA1c: Lopez-Cano et al. [15] reported that the expression of CLOCK, BMAL1, CRY1, CRY2, PER1-PER3, and RORA was significantly reduced in T2DM patients versus controls (all p < 0.05). Clock-gene expression correlated negatively with FPG, HbA1c, insulin, and HOMA-IR, and both HbA1c and clock-gene expression independently predicted HIF-1α expression.

Broader circadian gene landscape: The narrative review by Grskovic and Korac [14] reported that CRY2 variants are among the most consistently replicated for elevated fasting glucose across European and Asian populations, that MTNR1B is associated with impaired fasting glucose and beta-cell dysfunction, and that BMAL1 variants have been linked to both T2DM risk and insulin resistance; PER variants showed more heterogeneous associations.

Direction-of-Effect Summary

To make the direction and consistency of associations explicit under the SWiM framework, findings are categorised in Table 4 as a positive association (↑ higher glucose/insulin resistance/risk), reduced expression accompanying worse glycaemia (↓ expression), or a null/non-significant association (↔).

**Table 4:** Direction-of-effect summary across the included studies ↑, positive association with higher glucose/insulin resistance/T2DM risk; ↓ expression, reduced clock-gene expression accompanying worse glycaemia; ↔, null/non-significant association. CI, confidence interval; FPG, fasting plasma glucose; HOMA-IR, Homeostatic Model Assessment of Insulin Resistance; MetS, metabolic syndrome; OR, odds ratio; RERI, relative excess risk due to interaction

Gene / variant	Study	Outcome	Direction	Statistical significance
CLOCK rs1801260 × MTNR1A rs2119882	Li [10]	T2DM risk (interaction)	↑	Significant (RERI 1.07; 95% CI 0.23–1.91)
MTNR1B rs1387153	Li [10]	T2DM risk (night-shift)	↑	Significant (RERI 0.98; 95% CI 0.40–1.55)
CLOCK rs1801260 (single variant)	Li [10]	T2DM risk	↔	Not significant
CRY2 rs11605924 (recessive)	Ndonwi [11]	HOMA-IR	↑	Significant (p = 0.049)
CRY2 rs11605924 (recessive)	Ndonwi [11]	T2DM diagnosis	↑	Significant (OR 1.82; 95% CI 1.03–3.23)
CRY2 rs11605924 (recessive)	Ndonwi [11]	FPG	↑	Not significant (trend)
BMAL1 rs3789327 (GG)	Yang [12]	FPG	↑	Significant (β 0.101; 95% CI 0.003–0.199)
BMAL1 rs7950226 (A/AA)	Skrlec [13]	MetS incl. hyperglycaemia	↑	Significant (pooled; CI not reported)
CLOCK rs1801260 / rs6850524	Skrlec [13]	MetS risk	↔	Not significant (pooled)
CRY2 variants	Grskovic and Korac [14]	FPG (across studies)	↑	Consistent (narrative)
Core clock genes (expression)	Lopez-Cano [15]	HbA1c, FPG, HOMA-IR	↓ expression	Significant (all p < 0.05)

Risk of Bias

Of the six studies, two were rated low risk (Li et al. [10], NOS 7/9; Skrlec et al. [13], NOS 8/9) and four moderate risk (Ndonwi et al. [11], Yang et al. [12], Grskovic and Korac [14], and Lopez-Cano et al. [15]). Common sources of moderate risk were small primary-study samples [11,15], cross-sectional designs precluding causal inference [12,15], and the inherent selection bias of narrative review [14]. No study was rated high risk. Full domain scores are detailed in Table 5.

**Table 5:** Risk-of-bias assessment using the Newcastle–Ottawa Scale (NOS) ★ = criterion satisfied. Low risk: 7-9 stars; moderate: 5-6; high: ≤4. The adapted cross-sectional NOS (maximum eight stars) was applied to cross-sectional studies; for the systematic review/meta-analysis, the Newcastle-Ottawa Scale (NOS) was applied to the quality of the primary synthesis.

Author	Design	Selection: representativeness	Selection: ascertainment	Comparability	Overall (NOS)
Li et al. [10]	Case control	★	★	★★	Low (7/9)
Ndonwi et al. [11]	Case control	★	★	★	Moderate (6/9)
Yang et al. [12]	Cross-sectional	★	★	★	Moderate (6/8)
Skrlec et al. [13]	SR / meta-analysis	★	★	★★	Low (8/9)
Grskovic and Korac [14]	Narrative review	★	★	★	Moderate (6/9)
Lopez-Cano et al. [15]	Cross-sectional	★	★	★	Moderate (6/8)

Discussion

Summary of Findings

This review synthesised six studies (2020-2025) linking core circadian gene variants or expression to glycaemic parameters and insulin resistance in human populations with, or at risk for, T2DM [10-15]. Across heterogeneous designs, the evidence was directionally consistent: a CLOCK × MTNR1A interaction, CRY2 rs11605924, BMAL1 rs3789327, and global downregulation of clock genes were each associated with higher HOMA-IR, higher FPG, or worse HbA1c, supporting an association between molecular clock variation and impaired glucose homeostasis.

Mechanistic and Clinical Interpretation

CLOCK variants and gene-environment context: The finding by Li et al. [10] that CLOCK rs1801260 acts not in isolation but interacts additively with MTNR1A rs2119882 (RERI = 1.07) underscores that single-variant analyses may underestimate risk when epistatic and gene-environment effects are ignored. Consistent with a context-dependent CLOCK effect, Uemura et al. [16] reported that a CLOCK variant and related haplotypes were associated with T2DM prevalence in a Japanese population, and chrononutrition studies indicate that the glycaemic impact of clock variants is modifiable by meal timing and macronutrient composition [7]. This context dependence plausibly explains the null independent CLOCK rs1801260 result in the steelworker cohort once the MTNR1A interaction term is omitted.

CRY2 variants and insulin resistance: The CRY2 rs11605924 association with elevated HOMA-IR (p = 0.049) and T2DM risk (OR = 1.82) reported by Ndonwi et al. [11] is notable because South African mixed-ancestry cohorts are severely underrepresented in circadian genetics. The directional effect replicates earlier European GWAS identifying glucose-raising CRY2 polymorphisms [6]. Mechanistically, CRY proteins repress gluconeogenic gene expression and modulate hepatic glucocorticoid signalling; loss of CRY function elevates hepatic glucose output independently of pancreatic secretion, aligning with the observed elevation of HOMA-IR without proportionate FPG change.

BMAL1 variants and fasting glucose: The BMAL1 rs3789327 association with higher FPG in children [12], amplified by adiposity, is biologically plausible: BMAL1 drives rhythmic expression of genes governing adipogenesis, lipogenesis, and glucose uptake, and its disruption promotes beta-cell dysfunction and impaired glucose-stimulated insulin secretion in experimental models [3]. Marcheva et al. [17] further showed that alternative splicing under circadian control regulates beta-cell exocytosis and glucose homeostasis, and Rakshit and Matveyenko demonstrated that BMAL1 induction in beta-cells protects against obesity-induced glucose intolerance [18]. The meta-analytic association of BMAL1 rs7950226 with metabolic syndrome [13] consolidates BMAL1 as the circadian gene with the most consistent human metabolic evidence.

Global clock-gene expression and glycaemic control: Lopez-Cano et al. [15] showed that reduced expression of the entire core clock machinery correlates with worse FPG, HbA1c, and HOMA-IR, and that HbA1c and clock-gene expression independently predict HIF-1α. This suggests a bidirectional relationship in which poor glycaemic control further suppresses clock-gene expression, potentially creating a self-amplifying cycle of chronometabolic deterioration, implying that improving glycaemic control might partially restore clock-gene expression [19].

Comparison With Prior Literature

These results extend earlier work. The pilot study by Kelly et al. [20] reported nominal CRY2 and PER2 associations with T2DM in South Asian and European cohorts, directionally consistent with the present findings. Sookoian et al. [21] linked CLOCK variants to obesity, an important confounder not uniformly controlled across the included studies. Chrononutrition research by Garaulet and colleagues indicates that meal timing and clock-related genotypes jointly influence metabolic outcomes [7,18]. The melatonin receptor gene MTNR1B, although not a core clock gene, is a key input pathway linking light and melatonin rhythms to insulin-secretion timing, with variants associated with impaired fasting glucose across populations [10,14]. Differentiating evidence types, the disease-specific primary studies [10-13,15] carry greater weight for metabolic-pathway claims than the broad narrative review [14], which is best used for contextual framing rather than as primary support.

Clinical Implications and Therapeutic Targets

These observational findings suggest potential, rather than established, value for circadian information in diabetes care. Two applications are conceivable but require prospective validation. First, circadian genotyping could contribute to risk stratification, identifying carriers of glucose-raising clock variants for intensified prevention [22]. Second, chronotherapy - aligning meal timing, light exposure, sleep, and drug administration with endogenous rhythms - may offer a non-pharmacological route to improved glycaemic control, particularly in risk-allele carriers and shift workers [7,18].

Several pharmacological strategies that target the clock are under preclinical investigation and are noted here as future directions rather than current evidence: melatonin and its analogues (acting via MTNR1A/MTNR1B), REV-ERB agonists, and modulators of the energy-sensing NAD+-SIRT1-NAMPT axis that couple metabolism to the clock [5]. The flavonoid Nobiletin, a RORα/REV-ERB modulator, has shown clock-enhancing and metabolic effects in animal models. None of these agents has established efficacy for glycaemic control in humans, and they should be regarded as investigational [14].

Strengths

Strengths include strict PRISMA 2020 adherence, a comprehensive four-database search, independent dual-reviewer screening and quality appraisal, PICOS-defined eligibility, a structured SWiM direction-of-effect synthesis, and inclusion of ethnically diverse populations spanning East Asian, African, and European samples, including one of the first circadian-genetic datasets from a sub-Saharan African cohort.

Limitations

Several limitations apply. The small number of eligible studies (n = 6) reflects the genuinely limited volume of high-quality primary research directly linking circadian gene variants to quantitative glycaemic parameters in confirmed T2DM. Heterogeneity in designs, populations, genotyping platforms, and outcomes precluded meta-analysis and restricted synthesis to narrative methods, so causality cannot be inferred - the included case-control, cross-sectional, and review designs support association only, not causation. Ethnic representation was incomplete, with no eligible Middle Eastern or Latin American cohorts, limiting generalisability. Important confounders - chronotype, sleep quality, shift-work history, diet, and T2DM pharmacotherapy (metformin, SGLT-2 inhibitors, and GLP-1 receptor agonists) - were inconsistently reported. Restriction to English-language publications may have introduced language bias; publication bias favouring positive findings cannot be excluded, and the review was not prospectively registered.

Future Directions

Future research should prioritise adequately powered, multi-ethnic studies, explicitly including Middle Eastern and Latin American populations, using Mendelian randomisation and biobank-scale designs to test causality, longitudinal cohorts with objective chronotype and sleep phenotyping, and randomised trials of chronotherapy and clock-targeting agents. Standardised reporting of effect estimates with confidence intervals and consistent covariate adjustment would enable future quantitative synthesis.

## Conclusions

Core circadian gene variants and reduced clock-gene expression, spanning CLOCK, BMAL1, CRY2, and MTNR1B and their interactions, are consistently associated with higher FPG, HOMA-IR, HbA1c, and T2DM risk across diverse populations. Gene-gene (CLOCK × MTNR1A), gene-environment (clock variants × diet and adiposity), and global clock downregulation collectively implicate the molecular clock as a multilevel correlate of glycaemic homeostasis. Because the evidence is observational and heterogeneous, these associations suggest a potential value for circadian genetic profiling in diabetes risk stratification rather than established causal utility. Adequately powered, multi-ethnic longitudinal and Mendelian-randomisation studies with comprehensive chronophenotyping are needed before clinical implementation.

## Appendices

Appendix A: database-specific search strings

PubMed/MEDLINE: (“circadian clock”[MeSH] OR “CLOCK gene” OR “BMAL1” OR “ARNTL” OR “CRY1” OR “CRY2” OR “PER2” OR “PER3” OR “NR1D1” OR “REV-ERBα” OR “MTNR1B” OR “MTNR1A” OR “clock gene variant” OR “circadian gene polymorphism”) AND (“type 2 diabetes mellitus”[MeSH] OR “T2DM” OR “insulin resistance”[MeSH] OR “HbA1c” OR “fasting glucose” OR “HOMA-IR” OR “glycaemic control”) AND (“single nucleotide polymorphism”[MeSH] OR “SNP” OR “genotype” OR “variant” OR “polymorphism”).

Embase (Emtree): ('circadian rhythm'/exp OR 'CLOCK gene' OR 'BMAL1' OR 'ARNTL' OR 'cryptochrome' OR 'CRY1' OR 'CRY2' OR 'period circadian protein' OR 'NR1D1' OR 'MTNR1B' OR 'MTNR1A' OR 'clock gene variant':ti,ab) AND ('non insulin dependent diabetes mellitus'/exp OR 'T2DM' OR 'insulin resistance'/exp OR 'glycemic control' OR 'HbA1c' OR 'HOMA-IR':ti,ab) AND ('single nucleotide polymorphism'/exp OR 'SNP' OR 'genotype' OR 'genetic polymorphism'/exp).

Web of Science (Core Collection, TS=): TS=((“circadian clock” OR “CLOCK gene” OR “BMAL1” OR “ARNTL” OR “CRY1” OR “CRY2” OR “PER2” OR “PER3” OR “NR1D1” OR “MTNR1B” OR “MTNR1A”) AND (“type 2 diabetes” OR “T2DM” OR “insulin resistance” OR “HbA1c” OR “fasting glucose” OR “HOMA-IR”) AND (“polymorphism” OR “SNP” OR “genotype” OR “variant”)).

Cochrane Library (CENTRAL): (“circadian” OR “CLOCK” OR “BMAL1” OR “CRY2” OR “MTNR1B”) AND (“type 2 diabetes” OR “insulin resistance” OR “HbA1c”) AND (“polymorphism” OR “SNP” OR “variant”), searched in Title/Abstract/Keyword.

Appendix B: data extraction form

First author; publication year; country; study design; target population and ethnicity; sample size (cases/controls); T2DM diagnostic criteria; circadian gene(s) and specific variant(s); genotyping method; glycaemic and insulin-resistance outcomes assessed; statistical analysis approach; effect estimates (OR/β/RERI); 95% confidence intervals; p-values; reported covariate adjustments; and NOS risk-of-bias domain scores.
